# Update on diagnostic and therapeutic challenges of central hypothyroidism among childhood cancer survivors

**DOI:** 10.1007/s40618-025-02659-5

**Published:** 2025-07-28

**Authors:** Alice Casiraghi, Alessandro Cattoni, Luca Persani

**Affiliations:** 1 Department of Medical Biotechnology and Translational Medicine, University of Milan, Milan, Italy; 2 Department of Pediatrics, Fondazione IRCCS San Gerardo dei Tintori, Monza, Italy; 3 School of Medicine and Surgery, University of Milano-Bicocca, Monza, Italy; 4 Department of Endocrine and Metabolic Diseases, IRCCS Istituto Auxologico Italiano, Milan, Italy

**Keywords:** Childhood cancer survivors, Central hypothyroidism, Antineoplastic agents, Thyroid hormones, Quality of life, Endocrine disorders.

## Abstract

**Context:**

Following the exposure to toxic therapeutic agents employed in the treatment of malignancies, endocrine complications can affect up to 40–60% of childhood cancer survivors (CCS), with central hypothyroidism (CeH) being a relevant adverse event observed in this population. Given the long-standing uptrend in the number of CCS due to advances in antineoplastic and support therapies, the issue of treatment-related endocrine disorders, including CeH, has become an increasingly relevant topic.

**Methods:**

Pubmed search carried out using an ad-hoc query string in January 2025.

**Main results:**

The pathophysiology of CeH in CCS is primarily related to damage to the hypothalamic-pituitary-thyroid axis, often caused by the synergetic detrimental effect of antineoplastic treatments. Understanding the pathogenesis and the specificities of iatrogenic CeH is crucial to improve the clinical management of CCS. Due to the often subtle and paucisymptomatic nature of CeH in its early stages, regular, life-long thyroid screening is essential to prompt timely diagnosis. Indeed, early identification of CeH allows for appropriate thyroid hormone replacement, which is crucial for preventing long-term metabolic and developmental complications and to improve the quality of life of these patients.

**Conclusions:**

This review aims to enhance awareness among healthcare providers regarding the critical importance of timely detection of CeH and its specificities among CCS, in order to better understand the pathogenesis of treatment-related CeH and to outline evidence-based strategies for the diagnosis and treatment of CeH in this vulnerable population.

## Introduction

Over the last fifty years, significant advancements in diagnostics, pharmacology, treatment modalities, and support therapy have resulted in marked improvements in childhood cancer survival rates. Based on theoretical epidemiological projections, the number of cancer survivors is expected to achieve 26 million by the early 2040 s [1].

Accordingly, the scientific community has shed light over the increasing burden of late adverse effects experienced by cancer survivors as late as decades following the end of antineoplastic treatment [2].

This topic is especially relevant when addressing patients diagnosed with cancer and treated during childhood, as they have many years of life ahead of them. Hence, the timely detection of late adverse events is generally achieved by enrolling patients in life-long monitoring programmes, meant to be undertaken soon after the end of treatments and tailored with reference to the type of malignancy, therapy regimens, patient’s age upon diagnosis, and any pre-existing health conditions.

Endocrine complications affect 40–60% of childhood cancer survivors (CCS). Exposure to toxic therapeutic agents significantly increases the risk of disorders involving endocrine organs, including the hypothalamic-pituitary area, thyroid, and gonads. Iatrogenic endocrine disorders are associated with a considerable negative impact on both physical health and psychosocial well-being, resulting in a notable decline in overall quality of life [3].

Central hypothyroidism (CeH) is a common finding among CCS. The recent development of innovative treatment approaches and less invasive treatment protocols prompts the need for providing endocrinologists with a systematic update on incidence, diagnosis, and management of this condition.

CeH is characterized by impaired thyroid hormone production resulting from inadequate stimulation of the thyroid by decreased circulating levels of thyrotropin (TSH), despite a structurally intact gland [4].

The present analysis is meant to provide insights about the treatment-related pathogenesis of CeH among CCS, diagnostic challenges, and treatment recommendations.

## Methods

A systematic review was conducted using PubMed to identify previously published guidelines, original articles, clinical trials, systematic reviews and meta-analyses. The search was carried out using the following query string in January 2025: [(central hypothyroidism) OR (secondary hypothyroidism)] AND [(cancer survivors) OR (cancer treatment) OR (brain radiotherapy) OR (chemotherapy) OR (immune checkpoint inhibitors) OR (haematopoietic stem cell transplantation) OR (bexarotene) OR (mitotane)].

The search strategy incorporated highly specific terms such as “bexarotene” and “mitotane” given the well-established evidence of their significant role in the development of central hypothyroidism among patients treated for certain type of malignancies (see the specific sections).

Each article was reviewed for relevance, resulting in a final selection of 62 articles. The following exclusion criteria were applied to the research results: irrelevant case reports or manuscripts with language barriers.

The references of the included articles were reviewed and additional relevant articles were integrated.

The search was limited to the last 30 years.

### Central hypothyroidism among childhood cancer survivors: old and new determinants

Table 1 reports a summary of the agents playing a detrimental action on TSH secretion, relative pathogenesis, occurrence rates and specificities.

**Table 1:** Antineoplastic agents playing a detrimental role on TSH secretion thus leading to central hypothyroidism

Cancer treatment	Pathogenesis of tsh deficiency	Occurrence rates	Notes
Brain photon radiotherapy (phRT)	Progressive damage that develops over several years due to direct injury to hypothalamic neurons and vasculature, with secondary pituitary atrophy.	For doses ≥ 30 Gy, incidence is 3–9% among survivors of non-pituitary brain tumours [5]	Patients exposed to craniospinal or total body irradiation are at greater risk of combined primary and secondary hypothyroidism
Brain proton radiotherapy (pRT)	Progressive damage that develops over several years due to direct injury to hypothalamic neurons and vasculature, with secondary pituitary atrophy.	Conflicting outcomes by different sources: - trend towards decreased risk of both primary (7.3% after pRT, 22% after phRT) and central hypothyroidism (9.8% versus 24.0%) (10). - no statistically significant differences in the occurrence of central hypothyroidism following pRT versus phRT [13]	Further studies are needed to investigate longer-term effects of pRT
Immune Checkpoint Inhibitors (ICI)	Disruption of the protective mechanism of immune tolerance leading to autoimmune hypophysitis	Hypophysitis is observed in up to 10% of patients treated with anti-CTLA4-and 0.5-1% of patients receiving anti-PD-1 or anti-PD-L1 monotherapy [21]. Among patients with hypophysitis, TSH deficiency is the most frequent alteration (77–100%) [22].	MRI can be used to investigate the presence of pituitary alterations, however 23–33% of patients may not show abnormalities. For grade 1 and 2 hypophysitis, T4 treatment should be started without discontinuation of ICI therapy. For grade 3 hypophysitis, ICI therapy should be temporarily withheld, for grade 4 ICI therapy must be halted, and high-dose glucocorticoid therapy should be administered immediately
Haematopoietic stem cell transplantation (HSCT)	TBI affects the entire hypothalamic-pituitary axis and the thyroid, potentially disrupting any part of the TRH-TSH-thyroid feedback loop	Studies excluding patients who had prior cranial radiotherapy before TBI have reported a cumulative incidence of central hypothyroidism as low as 0% [28, 29]	
Bexarotene	reduction of TSHβ gene transcription, thereby impairing TSH secretion from thyrotrope cells activation of liver metabolic oxidative enzymes and glucuronidation, resulting in an increase of thyroid hormone metabolic clearance	Incidence of 29–100% during the treatment [32]	Testing of free T4 and TSH should be repeated 2–4 weeks after initiating bexarotene therapy and after every dosage modification. Bexarotene-related CeH is generally reversible
Mitotane	Combined impairment of TSH secretion and increase of TBG levels associated with competition on thyroxine TBG-binding sites	Incidence of 45.4% of patients within 3–6 months of treatment [45]	After mitotane discontinuation, most patients achieve euthyroidism in a period of 1–5 years

#### Brain photon radiotherapy (phRT)

Brain phRT has been extensively assessed as a known detrimental factor for TSH secretion.

High doses (up to 60 Gy) are administered for pituitary tumours, non-pituitary brain tumours, head and neck cancers (including nasopharyngeal carcinoma and rhabdomyosarcoma), and skull base tumours, whereas lower doses are delivered in patients with acute lymphoblastic leukaemia (ALL) and for total body irradiation as part of the conditioning regimen prior to hematopoietic stem cell transplantation (HSCT). Brain phRT can lead to neuroendocrine dysfunction when the radiation field includes the hypothalamic–pituitary area, with occurrence rates exceeding 80% of patients undergoing such therapy. The prevalence, time elapsed following iatrogenic trigger, and severity of the clinical pictures experienced are influenced by factors such as the total radiation dose, the number and magnitude of each dose fraction, the span of time elapsed between subsequent fractions, the age upon the time of irradiation and the overall duration of follow-up [5].

The selective radiosensitivity of each hypothalamic-pituitary axis determines the occurrence of variable hormonal deficiencies, with a well-defined gradient that ranges from growth hormone (GH - the most radiosensitive, with GH deficiency occurring at doses ≥ 18 Gy) to gonadotropin, ACTH, and TSH deficiencies (with this latter reported as the most resistant). In detail, TSH deficiency is typically observed following hypothalamic doses greater than 30 Gy, with a long-term cumulative incidence as high as 3–9% among survivors of non-pituitary brain tumours. Based on a mathematical model developed from the findings of 7 different study cohorts, Wheeler and colleagues proposes a 20%-risk of experiencing CeH following 22 Gy hypothalamic exposure divided into 2-Gy fraction [6]. However, the overall occurrence remarkably increases with longer-term follow-up and for doses exceeding 50 Gy [5].

Furthermore, patients exposed to craniospinal irradiation are at greater risk of combined primary and secondary hypothyroidism as the entire hypothalamic-pituitary-thyroid axis is involved in the radiation field, potentially causing impairment in any part of the regulatory pathway [7].

Hypothalamic-pituitary dysfunction resulting from cranial irradiation is a progressive phenomenon that develops over several years. The occurrence rate is affected by the radiation dose, with higher doses typically leading to earlier onset. Accordingly, a lifelong surveillance of hypothalamic-pituitary axis function is highly warranted among survivors [8]. 

Several pathophysiological mechanisms underlie radiation-induced hypopituitarism, but the nature of this phenomenon is still not fully understood. Direct injury to hypothalamic neurons and vasculature, with secondary pituitary atrophy, has been regarded as a potentially causative determinant. Microstructural changes within the hypothalamus, such as demyelination and axonal loss, and white matter lesions, which result from the obstruction of small cerebral vessels and subsequent hypoperfusion of the brain, contribute to this complex pathology [9].

### Brain proton radiotherapy (pRT)

The world of neuro-oncology has recently witnessed the outburst and development of new stereotactic radiation techniques, meant to empower effectiveness and to shrink adverse effects related to the over-exposure of healthy peri-neoplastic tissues. In particular, pRT has become the standard-of-care for children with diencephalic tumours candidate to cranial irradiation, mostly craniopharyngiomas, low grade gliomas and optic pathway gliomas. As pRT delivers radiations over a narrow range of depth, the irradiation field is shaped to minimize the exposure of healthy brain tissue [10]. Accordingly, it remarkably lowers the risk of late-onset radiation-related side effects compared to traditional photon-based technique.

Few studies have been conducted to compare the incidence of late endocrine effects following photon versus proton cranial radiotherapy in pediatric patients. Eaton and colleagues [11] found that pRT was associated with lower incidence of both primary and secondary hypothyroidism (23% vs. 69%, *p* < 0.001) and sex hormone deficiency (3% vs. 19%, *p* = 0.025), reduced need for endocrine replacement therapy (55% vs. 78%, *p* = 0.03), and greater height SDS at follow up. Concerning hypothyroidism, Bielamowicz and colleagues [12] highlighted a trend towards decreased risk of both primary (7.3% after pRT, 22% after phRT) and central hypothyroidism (9.8% versus 24.0%). Conversely, despite a sparing effect of pRT over non-target organs (such as the thyroid), no statistically significant differences in the occurrence of CeH following pRT versus phRT were outlined by Aldrich and colleagues, that recorded superimposable rates of TSH-, GH- and ACTH-deficiency [13]. However, further studies are needed to investigate longer-term effects of pRT.

Rose et al. proposed a reduced nocturnal TSH surge (< 50% of the average rise) as an additional criterium for the diagnosis of CeH after pRT and described CeH as a common sequela (up to 60%) of pRT, particularly for suprasellar and pharyngeal tumors [14].

### Chemotherapy

Available data on the incidence of CeH among CCS are mostly consistent with an overall neglectable detrimental effect of chemotherapy only regimens on TSH secretion patterns. Drawing data from a large cohort of leukaemic patients enrolled in the childhood cancer survivor study, Chow et al. highlighted that CeH is generally not observed in patients treated exclusively with chemotherapy [15]. In addition, van Santen demonstrated the lack of any degree of additional detrimental effect exerted by chemotherapy in a cohort of patients exposed to cranial, craniospinal, cervical, or thoracic radiation [16]. Conflicting results have been published by Rose and colleagues, who reported several cases of CeH out of 62 CCS treated with chemotherapy alone [14, 17]. This discrepancy may be explained by the diagnostic criteria employed by this latter study, as all the patients have been labelled as affected by CeH based on the response to TRH stimulation test or the blunted nocturnal TSH surge [18].

### Immune checkpoint inhibitors (ICIs)

Since their first approval by the Food and Drug Administration (FDA) in 2011, ICIs have rapidly become an integral part of many anti-cancer therapeutic regimens because of their efficacy in improving survival rates [19].

ICIs disrupt the protective mechanism of immune tolerance, facilitating the activation of antigen-specific T cells to target tumours [20]. However, this mechanism of action can simultaneously lead to a wide array of autoimmune reactions against self-organs, resulting in immune-related adverse events that can range from mild to life-threatening clinical pictures. In particular, autoimmune endocrinopathies are among the most common toxicities experienced by up to 40% of patients treated with ICIs [19].

Hypophysitis or autoimmune hypopituitarism is observed in up to 10% of patients treated with anti- Cytotoxic T lymphocyte-associated protein 4 (CTLA4)-based therapy. In contrast, it is less common in patients receiving anti-Programmed Death-1 (PD-1) or anti-PDLigand-1 (PD-L1) monotherapy, with an incidence of 0.5–1% [21]. Furthermore, while anti-CTLA-4 antibodies can cause combined pituitary deficiencies, anti-PD-1 or anti-PD-L1 antibodies are likely to cause central isolated adrenal deficiency.

Among patients with hypophysitis, TSH deficiency is the most frequent alteration (77–100%), followed by gonadotropin (55–100%) and corticotropin ones (50–88%) [22].

Manifestations usually become overt within six months following the start of ICI therapy; however, the timing of onset is highly variable and can occur at any point during treatment, even several months after therapy discontinuation. In patients treated with anti-CTLA4 antibodies, whether as monotherapy or in combination, symptoms generally appear within a median timeframe of 8 to 12 weeks after the beginning. On the other hand, patients receiving anti-PD-1/anti-PD-L1 antibodies typically exhibit symptoms later, with a median onset occurring between 24 and 26 weeks after the initiation of treatment [23].

Manifestations are related to hormonal deficiencies and, more rarely, to neurocompression due to oedema involving the suprasellar area.

Magnetic resonance imaging (MRI) can be used to investigate radiological pituitary alterations: findings may include pituitary enlargement, stalk thickening, and allogeneic or heterologous contrast enhancement in 77% of ICI-related hypophysitis cases, in particular in patients treated with anti-CTLA-4 antibodies rather than anti-PD-1/anti-PD-L1 antibodies [23]. However, 23–33% of patients may not show abnormalities on MRI, which is probably due to the timing of diagnostic imaging after recovery from swelling [23]. Therefore, even in the presence of normal MRI findings, ICI-induced hypophysitis cannot be ruled out and management should be based on clinical and biochemical evaluation. Close monitoring of symptoms, endocrinological biochemical findings, and a comprehensive evaluation are essential.

While resolution of central adrenal toxicity is rare, recovery from ICI-related CeH is quite a common occurrence [20].

Regarding the utility of high-dose corticosteroids in the treatment and resolution of ICI-related hypophysitis, given the lack of studies demonstrating a significant improvement in patient outcomes, guidelines recommend high-dose corticosteroids to be reserved only for patients with ICI-related hypophysitis who develop severe hyponatremia, significant mass effect from pituitary enlargement (e.g., compression of the optic chiasm), or those in a critical illness setting [20, 24].

For grade 1 (asymptomatic) or grade 2 (mildly symptomatic) immune checkpoint inhibitor-induced hypophysitis, hormone replacement can be initiated, and discontinuation of ICI therapy is not required. In cases of grade 3 (moderately symptomatic), ICI therapy should be temporarily withheld, and reintroduction of ICI treatment should be considered based on the patient’s clinical response to hormone replacement and glucocorticoid therapy. For grade 4 (severe or life-threatening), ICI therapy must be halted, and high-dose glucocorticoid therapy should be administered immediately [25].

### Haematopoietic stem cell transplantation (HSCT)

Over the past few decades, HSCT has become the cornerstone of treatment for a wide array of clinical conditions, including malignant and non-malignant haematological diseases, inherited metabolic disorders, and congenital immunodeficiencies [26]. In paediatric patients, endocrinopathies are a frequent late-onset complication, affecting more than 60% of long-term survivors who received HSCT before the age of 10 [27].

The conditioning regimen for HSCT is determined by clinical indications and patient-specific factors and may involve chemotherapy alone or a combination of chemotherapy and radiotherapy (total body irradiation, TBI). TBI consists of the administration of a uniform dose of ionizing radiation across the entire body to eliminate neoplastic cells and facilitate stem cell engraftment by suppressing immune rejection in the recipient [26]. TBI is a major contributor to post-transplant endocrinopathies, particularly when administered in a single dose (up to 10 Gy).

In contemporary protocols, TBI is typically delivered in three to nine fractions, with a total dose ranging from 10 to 16 Gy, to reduce the risk of complications [27]. Accordingly, CeH is rare following the low radiation doses used in TBI, as the TRH-TSH axis is highly resistant to radiation-induced damage. As previously mentioned, in cancer survivors the threshold for hypothalamic radiation exposure above which the risk of developing central hypothyroidism becomes clinically significant is generally considered to be 30 Gy. Studies excluding patients who had prior cranial radiotherapy before TBI have reported a cumulative incidence of CeH as low as 0% [28, 29]. Nevertheless, CeH has been observed in transplanted patients, particularly in those who have previously received cranial or craniospinal prophylaxis as part of first-line treatment for acute lymphoblastic leukaemia or central nervous system tumours [30]. This can be partially explained considering that during TBI the radiation affects the entire hypothalamic-pituitary axis and the thyroid, potentially disrupting any part of the TRH-TSH-thyroid feedback loop and finally resulting in a combined thyroid *plus* central dysfunction [31].

### Bexarotene

Bexarotene, a retinoid X receptor (RXR)-selective ligand, has been authorized by the FDA since 1999, as a second-line therapeutic option for early and late-stage refractory cutaneous T-cell lymphomas (CTCL). In Europe, it has been approved by the European Medicines Agency (EMA) for the management of advanced disease stages (IIB–IVB) [32]. CeH and hypertriglyceridemia are the two most common dose dependent adverse effects during treatment with Bexarotene, with a prevalence of 29–100% and 82–100% [32].

The relationship between thyroid function and vitamin A metabolism has been hypothesized for decades. Studies conducted in rats since the 1970 s have shown that a deficiency in vitamin A can lead to CeH, while vitamin A supplementation can reverse this effect by reducing TSH production and secretion [33].

In particular, experimental studies have demonstrated that RXR-selective agonists reduce TSH synthesis by suppressing TSHβ gene transcription, thereby decreasing TSH secretion from thyrotrope cells [34]. As a result, bexarotene induces pituitary hypothyroidism rather than hypothalamic hypothyroidism [35, 36]. Indeed, in a study conducted by Norikazu Toi et al., the TSH response to TRH stimulation testing was blunted one week after the initiation of bexarotene therapy, suggesting that bexarotene induces pituitary hypothyroidism in humans [37]. Furthermore, retinoids activate the liver metabolic oxidative enzymes as well as glucuronidation, resulting in an increase of thyroid hormone metabolic clearance [32].

Due to the high incidence of central hypothyroidism in patients treated with bexarotene, baseline testing, including measurement of serum TSH and free T4 levels, should be performed before starting patient on this medication. Baseline free T4 levels provide clinicians with a useful therapeutic target for subsequent monitoring and treatment. Testing of free T4 and TSH should be repeated 2–4 weeks after initiating bexarotene therapy and after every dosage modification. Bexarotene-related CeH is generally reversible, and tests performed one month after the discontinuation of this medication generally confirm the restoration of a fully retrieved thyroid function [38].

### Mitotane

Mitotane is a steroidogenesis inhibitor with adrenolytic and cytotoxic proprieties used to treat adrenocortical carcinoma. Some studies have reported mitotane-induced CeH [39, 40].

The causative mechanism leading to impaired TSH secretion remains unclear in this setting. In addition, mitotane has been reported to increase thyroxine binding globulin (TBG) levels and compete with thyroxine for TBG-binding sites [41, 42]. However, these findings do not fully explain the alterations in thyroid function seen recorded during treatment with mitotane. Other potential mechanisms, such as changes in deiodinase activity and depressed pituitary function, have also been proposed [43]. In studies using an in vitro murine pituitary cell model, mitotane was shown to suppress TSH secretion, inhibit TSH response to TRH, and induce apoptosis at therapeutic concentrations [44].

The onset of CeH more frequently occurs in the first year of treatment, with reported incidence rates achieving 45% within 3–6 months of treatment [45].

Following mitotane discontinuation, retrieved thyroid function tests are restored in most survivors. Nevertheless, as the recovery process can be slow, 1 to 5 years may elapse following mitotane discontinuation before euthyroidism is achieved [46].

These features emphasize the pivotal role of regular monitoring of thyroid function, including free T4 levels, both during and after the cessation of mitotane therapy [46].

## Diagnostic challenges

The diagnosis of CeH is generally based on the biochemical finding of the combination of low FT4 and low or inappropriately normal TSH levels, confirmed upon two sequential biochemical assessments.

Moreover, in patients screened for co-occurrent hypothalamic-pituitary disorders, mild forms of CeH should be suspected when serum FT4 levels decrease from higher values into the lower quartile of the reference range [4]. A 20%- or greater decrease in FT4 levels compared to baseline levels in the setting of low/normal TSH should be emphasized and prompt the clinical suspicion of CeH, as long as the tests are performed in the same laboratory and with the same assay method [4, 47]. Indeed, currently most FT4 and FT3 immunoassays exhibit substantial negative or positive biases that surpass the intra-individual biological variability. As a result, the reference ranges for FT4 and FT3 immunoassays are dependent on the specific assay method used [48]. Therefore, FT4 levels should be measured with the same assay in the same lab during the follow-up of CCS in order to reliably estimate a 20% decrement in circulating FT4 [4]. Such decrement can be supported by additional parameters of thyroid hormones action (e.g., cholesterol, heart rate, hypothyroidism questionnaire) or dynamic tests (e.g., TRH test or nocturnal TSH surge).

Signs and symptoms consistent with hypothyroidism should be periodically assessed. Endocrinologists should be aware that CCS may experience a combination of multiple hormonal deficiencies and non-endocrine conditions, which can interplay and lead to signs and symptoms that mimic hypothyroidism. In detail, hypogonadism, growth hormone deficiency, hypocortisolism, chronic cardiopulmonary disorders, as well as psychological issues and side effects of specific cancer therapies can all contribute to the onset of fatigue, weight gain, reduced physical activity tolerance, and depression [26].

Published guidelines recommend that surveillance for CeH is undertaken upon diagnosis in patients with CNS tumours involving the hypothalamic-pituitary area and/or in case of neurosurgical procedures affecting nearby anatomical structures [8, 49]. On the other hand, patients exposed to cranial RT should begin annual monitoring 6–12 months after the completion of radiotherapy.

In detail, patients exposed to hypothalamic-pituitary radiation doses ≥ 30 Gy should receive lifelong surveillance with at least annual measurement of TSH and FT4, given for the concrete risk of developing TSH deficiency.

There may be challenges in interpreting thyroid laboratory results, as CeH and primary thyroid dysfunction can overlap when the hypothalamus, pituitary, and thyroid gland have been subjected to radiation therapy, for example in craniospinal RT [49].

TRH test can play a supportive role in confirming the suspicion of mild CeH and differentiating tertiary (hypothalamic) from secondary (pituitary) hypothyroidism, as these conditions may present with exaggerated, delayed (peak after 60 min), or prolonged, versus blunted (< 4 mU/L) TSH responses to TRH 200 mcg i.v injection, respectively [50]. However, it is important to note that a significant proportion of patients with CeH may still show a normal TSH response after TRH stimulation test and a normal TRH test is not able to rule out CeH [51]. Additionally, distinguishing between these two forms of CeH can be challenging, because both the hypothalamus and pituitary can be affected in most patients. Therefore, the practical use of TRH test is primarily limited to patients with an uncertain diagnosis, where an abnormal TSH response may help confirm the presence of CeH [50].

### Treatment recommendations

The first-line replacement treatment for central hypothyroidism is represented by oral levothyroxine (L-T4) administered daily. The dosage of L-T4 should be tailored based on the patient’s weight, age, specific needs and co-occurrent medications, but a suggested starting dose is 1.6 mcg/kg/day [4, 52]. After 6–8 weeks of treatment, thyroid function tests should be prescribed in order to assess the adequacy of the dose prescribed [53]. In detail, endocrinologists should aim at keeping FT4 values above the median of the reference range. In patients with low TSH levels at diagnosis, monitoring TSH is not required during L-T4 replacement therapy. However, in patients with normal TSH levels at diagnosis, a lack of TSH suppression may suggest insufficient replacement [4, 53]. It is essential that blood samples for hormone measurements are withdrawn either before or at least 4 h after the daily L-T4 dose administration, in order to avoid FT4 over-estimation. In addition, thyroid function tests should be always performed in the same laboratory, to ensure analytic accuracy [53].

Importantly, L-T4 replacement therapy should be started only after excluding cortisol deficiency by performing a systematic assessment of adrenal reserve. Indeed, thyroid hormones prompt endogenous cortisol clearance and metabolism, thus leading to increased glucocorticoid requirement [52]. Accordingly, correction of hypothyroidism may precipitate adrenal crisis in case of co-occurrent ACTH deficiency in patients at risk for multiple hypothalamic-pituitary disorders [52]. In case of impaired adrenal reserve, glucocorticoid replacement therapy should be safely started few days before L-T4 to prevent signs and symptoms of adrenal insufficiency [4, 49].

In patients with multiple pituitary hormone deficiencies, it is also crucial to consider additional endocrine confounders. Both estrogens and GH play a biological role on thyroid hormones transportation and metabolism and can mask an underlying CeH [52]. In detail, the introduction of recombinant human GH (rhGH) replacement therapy in patients with GH deficiency (GHD) or the increase of serum estrogens levels often result in a decrease of FT4 levels [47, 54, 55]. This evidence suggests that untreated GHD or female hypogonadism can mask partial or subclinical CeH.

Consistently, patients already on treatment with levothyroxine generally show an increase in the needed dose after either estrogen or rhGH replacement therapy are started [56].

Therefore, close and continuous monitoring of thyroid function tests is essential when managing children with combined pituitary hormone deficiencies, especially when prescribing rhGH or estrogen therapy [56].

Furthermore, patients exposed to neurosurgery or cranial RT at risk for TSH deficiency are also prone to experience seizures or epilepsy. Antiepileptic drugs, such as phenytoin, valproate, carbamazepine, oxcarbazepine and topiramate promote thyroid hormones metabolism and affect TSH and FT4 transportation [57]. Accordingly, clinicians should closely monitor thyroid hormone levels when initiating or adjusting the dosage of antiepileptic medications [8].

Table 2 provides a summary for the endocrine and antiepileptic medications affecting TSH secretion and thus potentially involving or precipitating a picture consistent with CeH.

**Table 2:** Summary of the effects of hormonal and antiepileptic medications over TRH-TSH-thyroid axis

Treatment	Mechanism of interaction with thyroid axis	Clinical recomendations
rhGH	rhGH replacement therapy in GHD patients causes a decrease in fT4 levels within 3–6 months from the start of GH therapy, likely due to an increased metabolism [52]	Clinicians should monitor GHD patients for developing CeH after they start or adjust GH therapy [52]
Estrogens	Estrogens induce an increase in TBG serum concentration, thus requiring an increment of thyroid stimulation which cannot be achieved in patients with combined pituitary defects [54]	In patients with CeH requiring changes in estrogen therapy, it is important to monitor fT4 levels and adjusting L-T4 doses to maintain fT4 levels within target ranges [52]
Androgens	Androgens reduce TBG serum concentration	CeH patients receiving androgens do not require L-T4 dose adjustments
Glucocorticoids (GCs)	High GCs doses can transiently suppress TSH secretion. Thyroid hormone accelerates endogenous cortisol clearance and metabolism, leading to increased glucocorticoid requirement [52]	It is pivotal to exclude cortisol deficiency or give hydrocortisone supplementation before introducing L-T4 therapy. Conversely, CeH patients receiving GC therapy do not require L-T4 dose adjustments [52]
Phenytoin	Competitively reduces the binding of T4 to TBG [58] Potent inducer of hepatic microsomal enzymes, particularly CYP enzymes [59] Induction of glucuronosyltransferases [60]	Therapeutic levels of phenytoin can displace T4 and T3 from serum binding proteins, potentially leading to falsely low free T4 concentrations in commercially available assays. Therefore, it is crucial to place significant emphasis on clinical manifestations when adjusting thyroid hormone therapy [57]. Because of the induced hepatic metabolism, phenytoin may require an adaptation of L-T4 replacement and may uncover a latent thyrotrope insufficiency
Phenobarbital	Competitively reduces the binding of T4 to TBG (weak effect)[61] Potent inducer of hepatic microsomal enzymes, particularly CYP enzymes [59] Induction of glucuronosyltransferases (minimal effect on thyroid hormone homoeostasis) [60]	Because of the induced hepatic metabolism, phenobarbital may require an adaptation of L-T4 replacement and may uncover a latent thyrotrope insufficiency
Carbamazepine/Oxcarbazepine	TSH suppression Marked TBG inhibition [62] Induction of CYP3A4 and partial induction of UGT [60]	TSH suppression may uncover a latent thyrotrope insufficiency. Therapeutic levels of these drugs can lead to falsely low free T4 concentrations in commercially available assays. Therefore, it is crucial to place significant emphasis on clinical manifestations when adjusting L-T4 therapy [57].
Valproate	Patients receiving valproate therapy have an increased risk for mild primary hypothyroidism	CeH patients receiving valproate rarely require L-T4 dose adjustments

rhGH– recombinant human growth hormone; CeH– central hypothyroidism; GC– glucocorticoids; UGT - uridine diphosphate (UDP)-glucuronosyltransferase; TBG– thyroxine binding globulin

Cancer survivors experience a higher incidence of metabolic complications, such as diabetes and obesity, compared to otherwise healthy controls. Cancer treatments, including high-dose glucocorticoids, can contribute to weight gain and disrupt metabolic parameters such as glucose and lipid levels. In addition to common risk factors for overweight, such as poor diet, physical inactivity, and genetic predisposition, certain cancer treatments like cranial radiation and alkylating chemotherapy, can lead to endocrine dysfunctions, including GHD, hypogonadism, and hypothyroidism, which further elevate the risk of weight gain. These metabolic disturbances are key features of the metabolic syndrome, which is associated with increased cardiovascular disease risk and mortality [3]. Given the critical role played by thyroid hormones in growth, development, fatigue, weight or body mass index, waist circumference, and cholesterol levels, it is crucial to start adequate LT4 treatment even in mild defects of TSH secretion [52].

## Conclusion

In conclusion, cancer survivors represent an increasingly vulnerable population worldwide. They are susceptible to long-term complications that may emerge years or even decades following cancer treatment, potentially affecting quality of life. Life-long, individualized follow-up is critical for the early detection and management of endocrine disorders, ensuring timely interventions that are particularly important given the essential role of thyroid hormones in growth and development. Early and tailored endocrine surveillance may significantly reduce long-term morbidity and alleviate the burden on survivors, caregivers, and healthcare systems.

CeH is a non-infrequent late effect mostly experienced by brain cancer survivors. Due to its underhand or indolent presentation, clinicians should be trained to identify early signs of this condition, in order to prevent survivors from experiencing long-lasting untreated symptoms and comorbidities.
